# From 2 dimensions to 3^rd^ dimension: Quantitative prediction of anterior chamber depth from anterior segment photographs via deep-learning

**DOI:** 10.1371/journal.pdig.0000193

**Published:** 2023-02-01

**Authors:** Zhi Da Soh, Yixing Jiang, Sakthi Selvam S/O Ganesan, Menghan Zhou, Monisha Nongiur, Shivani Majithia, Yih Chung Tham, Tyler Hyungtaek Rim, Chaoxu Qian, Victor Koh, Tin Aung, Tien Yin Wong, Xinxing Xu, Yong Liu, Ching-Yu Cheng

**Affiliations:** 1 Singapore Eye Research Institute, Singapore National Eye Centre, Singapore; 2 Yong Loo Lin School of Medicine, National University of Singapore, Singapore; 3 Institute of High Performance Computing, Agency for Science, Technology and Research, Singapore; 4 Ophthalmology & Visual Sciences Academic Clinical Program, Duke-NUS Medical School, Singapore; 5 Department of Ophthalmology, The First Affiliated Hospital of Kunming Medical University, Kunming, China; 6 Department of Ophthalmology, National University Hospital, Singapore; 7 Tsinghua Medicine, Tsinghua University, China; University of Cagliari: Universita degli Studi Di Cagliari, ITALY

## Abstract

Anterior chamber depth (ACD) is a major risk factor of angle closure disease, and has been used in angle closure screening in various populations. However, ACD is measured from ocular biometer or anterior segment optical coherence tomography (AS-OCT), which are costly and may not be readily available in primary care and community settings. Thus, this proof-of-concept study aims to predict ACD from low-cost anterior segment photographs (ASPs) using deep-learning (DL). We included 2,311 pairs of ASPs and ACD measurements for algorithm development and validation, and 380 pairs for algorithm testing. We captured ASPs with a digital camera mounted on a slit-lamp biomicroscope. Anterior chamber depth was measured with ocular biometer (IOLMaster700 or Lenstar LS9000) in data used for algorithm development and validation, and with AS-OCT (Visante) in data used for testing. The DL algorithm was modified from the ResNet-50 architecture, and assessed using mean absolute error (MAE), coefficient-of-determination (R^2^), Bland-Altman plot and intraclass correlation coefficients (ICC). In validation, our algorithm predicted ACD with a MAE (standard deviation) of 0.18 (0.14) mm; R^2^ = 0.63. The MAE of predicted ACD was 0.18 (0.14) mm in eyes with open angles and 0.19 (0.14) mm in eyes with angle closure. The ICC between actual and predicted ACD measurements was 0.81 (95% CI 0.77, 0.84). In testing, our algorithm predicted ACD with a MAE of 0.23 (0.18) mm; R^2^ = 0.37. Saliency maps highlighted the pupil and its margin as the main structures used in ACD prediction. This study demonstrates the possibility of predicting ACD from ASPs via DL. This algorithm mimics an ocular biometer in making its prediction, and provides a foundation to predict other quantitative measurements that are relevant to angle closure screening.

## Author summary

This proof-of-concept study aimed to predict anterior chamber depth (ACD) quantitatively from anterior segment photographs (ASPs) using deep-learning. Anterior chamber depth is a major and consistent risk factor of primary angle closure disease (PACD), which is a major cause of glaucoma-induced blindness at the later stages. Our study is motivated by the lack of an appropriate screening tool for PACD, where clinical tests lack repeatability and imaging devices are too costly to adopt in primary care and community settings for screening. In this study, we included 2,311 pairs of ASPs and ACD measurements in algorithm development, and 380 pairs for algorithm testing. We modified the ResNet-50 convolutional neural network (CNN) in developing our algorithm. Our algorithm was able to predict ACD with a mean absolute error of 0.18mm in algorithm validation, and 0.23mm in algorithm testing. We obtained an intraclass correlation coefficient (ICC) of 0.81 in validation, indicating good agreement between actual and predicted ACD measurements. Importantly, our algorithm highlighted the pupil and its margins in predicting ACD, which is similar to how actual measurements were obtained from imaging devices. Our study shows that depth (i.e., ACD) may be predicted from 2-dimension photographs using deep-learning. This algorithm provides a foundation for predicting other relevant parameters of PACD, which may be further combined into a screening algorithm for the disease at a later stage.

## Introduction

Primary angle closure disease (PACD) is a spectrum of disease that is characterized in common by an obstruction to aqueous humor outflow (e.g., irido-trabecular contact) and may culminate in the development of glaucomatous optic neuropathy. [1] Primary angle closure glaucoma (PACG) is the more visually debilitating form of glaucoma with high undetected rates across diverse communities in Asia. [2] A problem with PACG detection lies in its often asymptomatic nature, especially in the early stages. [3] Thus, to mitigate against vision loss in PACG, there is a need to detect angle closure in the community for regular monitoring and timely interventions. This is particularly important in Asia where PACD is more prominent and PACG is a major form of glaucoma. [4,5]

The lack of an appropriate screening tool to detect angle closure at the community level remains a problem. [2] Gonioscopy, the current gold standard for anterior chamber angle assessment, is a clinically demanding and time-consuming test that requires technical expertise, access to slit-lamp bio-microscope, and the application of local anaesthesia to perform. [6] Furthermore, gonioscopy assessment is subjective and known to have wide inter-assessor variability. [7,8] However, there is a lack of viable alternatives. Anterior segment optical coherence tomography (AS-OCT) can provide cross-sectional photo documentation for angle assessment but are bulky, expensive, and impractical for community screening. [9] In contrast, methods such as the Van Herick or the oblique flashlight test, are quick and relatively easy to perform but are associated with sub-optimal performances in detecting PACD. [10–12]

Nonetheless, the advents of artificial intelligence and deep learning (DL) in recent years have provided us with multiple opportunities for value innovation. In ophthalmology, DL is often utilized in image analysis for disease screening, [13,14] and regulatory approval has been obtained for its use in diabetic retinopathy screening in some countries. [15,16] Although, relatively fewer DL algorithms have been developed for anterior segment eye diseases, available studies shows that DL is able to effectively detect pterygium from anterior segment photographs, [17] and angle closure from AS-OCT scans, [18] thereby highlighting the potential use of DL with different anterior eye imaging modalities.

Anterior chamber depth (ACD) is a major risk factor of angle closure that has been suggested for use as a quick screening parameter for PACD. [19] Studies reported ACD to be a strong determinant of angle width measurements obtained from AS-OCT [20] and correlates well with peripheral anterior synechiae (PAS) formation. [21] Moreover, ACD has been shown to be able to detect 81–90% of angle closure cases in different settings, including rural Taiwan [22] and Mongolia, [23] with sensitivity ranging from 76.4–83.0% and specificity ranging from 67.2–88.9% (**S1 Table**). Importantly, ACD is quick to administer in large scale screenings and it is also intuitively easier to comprehend and interpret as a screening parameter. [23,24] However, ocular biometer used for ACD measurements may not be readily available in primary care or community settings, which preclude its potential use for screening.

Hence, the present study aims to develop a proof-of-concept DL algorithm to predict ACD quantitatively from anterior segment photographs. The ability to demonstrate utility of this algorithm may further act as an important foundation for predicting other quantitative measurements that are relevant in screening for PACD.

## Methods

### Study population

This cross-sectional study was conducted at the Singapore Eye Research Institute (SERI) according to the Declaration of Helsinki after ethics approval was obtained from SingHealth Centralized Institutional Review Board. Written informed consent was obtained from all participants in the respective studies.

First, we included a sub-set of data from the Singapore Malay Eye Study (SiMES) in algorithm training and validation. The detailed methodology of SiMES has been described previously. [25] Briefly, age-stratified random sampling was used to select Malay adults aged 40 years and above from the south-west region of Singapore. Baseline examination was conducted from 2004–2006 (response rate 78.7%) with follow-up examinations conducted first in 2011–2013 and again in 2017–2019. We included data from the 2^nd^ follow-up examination (2017–2019) in this study.

Second, we included data from the Iris Surface Features (ISF) study, which is a cross-sectional clinical imaging study on PACD, in algorithm training and validation. [26,27] This study recruited participants aged ≥40 years who were diagnosed with PACD clinically and had a patent laser peripheral iridotomy in the affected eye, and further included a small number of participants with open angles as control. Participants with previous ocular surgery (e.g., cataract or glaucoma filtering surgery), history of penetrating injury, and excessive corneal opacity or extensive pterygium (defined as covering ≥50% of iris area) were excluded.

Next, we further included a sub-set of data from the Singapore Chinese Eye Study (SCES) and the Singapore Indian Eye Study (SINDI) for algorithm testing. These studies, along with SiMES, are part of the Singapore Epidemiology of Eye Diseases (SEED) study. [25] For SCES, baseline examination was conducted from 2009–2011 (response rate 72.8%) with follow-up examination conducted between 2015–2017. For SINDI, baseline examination was conducted from 2007–2009 (response rate 75.6%). We included data from the 1^st^ follow-up examination in both SCES and SINDI as the 2^nd^ follow-up examination is currently ongoing.

### Anterior segment photography

Anterior segment photographs were obtained according to a standardized protocol in SEED and the ISF study (**S1 Fig**). All assessors, who were trained optometrists, had to be validated by a co-investigator (SM) prior to data capture. A digital camera (DC3; Topcon Corporation, Tokyo, Japan) was mounted on a slit-lamp bio-microscope (Topcon Corporation, Tokyo, Japan) to obtain color photographs of the anterior eye at 16x magnification in a dark room (≤20 lux). We utilized diffuse illumination; slit beam at full width (20mm) and height (14mm); at 30% of maximum brightness without flash; tilted at approximately 45 degrees temporally. Participants were asked to look straight ahead and any decentration in gaze were manually mitigated by directing their line-of-sight. Images were captured with both the upper and lower eyelids retracted to expose the full cornea circumference and if possible, its surrounding bulbar conjunctiva. To mitigate against variation in the above specifications, physical markings were placed on the slit-lamp bio-microscope to guide assessors. After image capture, quality checks were performed on a 1366x768/60-Hz resolution screen by the author (ZDS).

### Anterior chamber depth measurement

We measured ACD using non-contact partial coherence laser interferometry in SiMES (IOLMaster 700, Carl Zeiss Meditec AG, Jena, Germany), and optical low-coherence reflectometry in the ISF study (Lenstar LS900, Haag-Streit, Koeniz, Switzerland). The ACD values acquired from the IOLMaster included the central corneal thickness (CCT), while the ACD measurement from the Lenstar biometer automatically excluded CCT (aka “Aqueous depth”). To be consistent, in the present study we defined ACD as the distance along the visual axis from the corneal endothelium to the anterior crystalline lens surface. Therefore, we calculated the “true” ACD values in SiMES by subtracting CCT from ACD values obtained from IOLMaster. [28] In both studies, participants were asked to blink normally just before measurements to mitigate against dry-eye related errors in measurements. We took 3 measurements manually with the Lenstar biometer, whereas the IOLMaster automatically produced five simultaneous measurements each time. We utilized the inbuilt quality assessment function in both biometers to appraise the quality of measurements taken. The mean ACD value was recorded for use if all measurements had good quality checks or were otherwise repeated (up to 2 further attempts).

In SCES and SINDI, ACD measurements were obtained from anterior segment optical coherence tomography (Visante AS-OCT, Carl Zeiss Meditec AG, Jena, Germany). The first author (ZDS) manually annotated the scleral spurs (SS) with the Zhongshan Angle Assessment Program (ZAAP) after good inter-assessor agreement (intraclass correlation coefficients: 0.999 [x-axis]; 0.956 [y-axis]) was obtained with a senior author (MN). The definition of SS used has been described previously. [29] We took the exact ACD values obtained from ZAAP as it does not include CCT values after appraisal of structure segmentation.

### Angle closure assessment

In all included studies, gonioscopy was performed according to a standardized protocol by ophthalmology-trained research fellows using a Goldmann two-mirror contact lens (Ocular Instruments Inc., Bellevue, USA) under standard dark illumination. The detailed methodology of gonioscopy has been described previously. [30] Angle closure was diagnosed in eyes where the posterior trabecular meshwork (PTM) was not observed in 2 or more quadrants (i.e., ≥180 degrees) on gonioscopy.

### Algorithm development and testing

We included participants who were phakic and had both anterior segment photographs and ACD measurements. We excluded anterior segment photographs with dim illumination, eyelids blockage, decentered gaze, motion artifacts and poor focus covering more than one-third of the iris. We further excluded ACD measurements with poor quality or fixation error.

We adopted and modified the Residual Network 50 (ResNet50) architecture, which was originally trained on the ImageNet dataset that comprised of 1.28 million images over 1000 object classes, in this study. [31] The ResNet-50 architecture is a widely used convolutional neural network that comes with a well fine-tuned training setting, [32] and is more efficient in terms of computation and accuracy trade-off as compared to the ResNet-34. [31] In addition, the ResNet-50 was similarly adopted in previous studies that predicted quantitative measurements from fundus photographs. [33,34] Modifications were made to the original architecture prior to ACD prediction (**S2 Fig**). First, we replaced the fully connected layer of ResNet-50, which was developed for classification task, with a linear layer to derive at a single continuous output (i.e., ACD measurement). The activation function (ReLu) was removed after the linear layer. Second, the weights of the first convolutional layer were reinitialized using He initialization. By including HE initialization in the first layer, the algorithm may be able to learn low-level features that are specific to our dataset with higher efficiency. [35]

We utilized Open-Source Computer Vision Library (OpenCV v4.5, Intel Corporation, California, USA) for image pre-processing. Images were first resized to (224, 224, 3), and image brightness was increased by 20%. Histogram equalization was then used for contrast enhancement, followed by an image normalization that scales the pixel values to zero mean and unit variance. After that, the processed images were used as inputs to the neural network was of size (224, 224, 3). In addition, data augmentation was performed during the training stage to mitigate against overfitting. Specifically, random rotation from -35 to 35 degrees, random horizontal flip with a probability of 0.5, and random vertical flip with a probability of 0.1 were used.

We paired each anterior segment photograph with its corresponding ACD measurement. Then, the overall dataset was randomly split 4:1 into a training and validation dataset. The batch size used was 32. We ensured both eyes of a participant were either in the training or validation dataset by randomly splitting image pairs at the participant level, which mitigates against biased evaluation of our algorithm. Random shuffling was used for algorithm training. PyTorch (Facebook’s AI Research lab, California, USA), an open-source software library for DL, was used in algorithm training and evaluation. [36] Specifically, the algorithm was trained using a RTX3090 GPU with PyTorch v1.9.0, CUDA 11.1 and cuDNN v8.1.0 installed. Transfer learning was adopted and pre-trained weights from ResNet-50 were used. The two modifications described earlier were applied after loading the pre-trained weights. Adam optimizer with a learning rate of 4e-4 was used to train the model for 200 epochs. The mean absolute error (MAE) was used as the loss function, and the evaluation metrics include MAE and coefficient of determination (R^2^).

To enhance the interpretability of our algorithm, the Gradient-weighted Activation Mapping (Grad-CAM) algorithm was used to generate saliency maps for the neural network. [37] Specifically, the last layer inside layer block 4 in ResNet-50 was used as the target layer. These maps highlighted important regions for the final prediction based on gradients. To get an aggregated visualization, saliency maps for individual images are normalized to [0,1] and averaged across images to get the averaged saliency map.

### Statistical analysis

The intraclass correlation coefficients (ICC) and Bland-Altman plot were used to evaluate the agreement between predicted and actual ACD measurements. The ICC is a reliability index that ranges from 0 to 1 and reflects both the degree of correlation and agreement between measurements. [38] We measured ICC using a single rater, two-way mixed-effects, absolute agreement model, and defined values <0·5 as poor, 0.5 to 0.75 as moderate, 0.75 to 0.90 as good, and >0.90 as excellent agreement. [39] In the Bland-Altman plot, the difference between two measurements was plotted against the average of the two measurements. [40] We evaluated systematic (fixed) bias by testing if the mean difference between the two measurements was significantly different from zero using a one-sample t-test. Then, proportional bias was evaluated by testing whether the slope of the least squares regression line in the Bland-Altman plot was significantly different from zero. This was tested with the Pearson’s correlation coefficient. [41,42] We further assessed the accuracy of predicted values with the mean absolute error (MAE) and R^2^. All statistics were performed using STATA version 17 (STATA Corp, Texas, USA) and SciPy package.

## Results

We included 1,738 eyes of 943 participants from SiMES, and 575 eyes of 343 participants from the ISF study in developing our DL algorithm (**S2 Table**). In SiMES, 6.3% of eyes included were diagnosed with angle closure, and had a mean (standard deviation) ACD of 2.56 (0.33) mm. In the ISF study, 92.7% of eyes included were diagnosed with angle closure, and had a mean ACD of 2.08 (0.32) mm. The range of ACD measurements was between 1.47 to 3.91 mm in SiMES and 1.50 to 3.59 mm in the ISF study. The training dataset comprised of 1,029 participants from SiMES and the ISF study (**Table 1**). This comprised of 1,849 eyes with a mean ACD of 2.44 (0.39) mm, of which 27.4% had angle closure. The range of ACD values included in the training dataset was normally distributed between 1.47 to 3.91mm (**S3 Fig**), of which 13.5% (n = 250 eyes) had ACD <2mm; 42.1% (n = 778 eyes) had ACD between 2 to <2.5mm; 36.7% (n = 678 eyes) between 2.5 to <3mm; 7.7% (n = 143 eyes) had ACD >3mm. The validation dataset included 462 eyes of 257 participants with a mean ACD of 2.43 (0.37) mm (range 1.57–3.85mm), of which 29.2% had angle closure.

**Table 1:** Demographic and ocular characteristics of participants included in this study.

	Training data	Validation data	Testing data
Participants (N)	1029	257	380
Age (years)	65.1 (7.9)	65.2 (8.3)	60.9 (7.2)
Gender (Male, %)	463 (45.0)	113 (44.0)	189 (49.7)
Ethnicity (Chinese, %)	252 (24.5)	64 (24.9)	229 (60.3)
Eyes (N)	1849	462	380
Angle status (%) • Open	1342 (72.6)	327 (70.8)	271 (71.3)
• Closed*	507 (27.4)	135 (29.2)	109 (28.7)
Anterior chamber depth (mm)	2.44 (0.39)	2.43 (0.37)	2.58 (0.36)

Data presented are mean (standard deviation) for continuous variables; frequency (percentage) for categorical variables

Footnote: *Angle closure was diagnosed in cases where ≥180° posterior trabecular meshwork was not observed with gonioscopy

**Fig 1:**
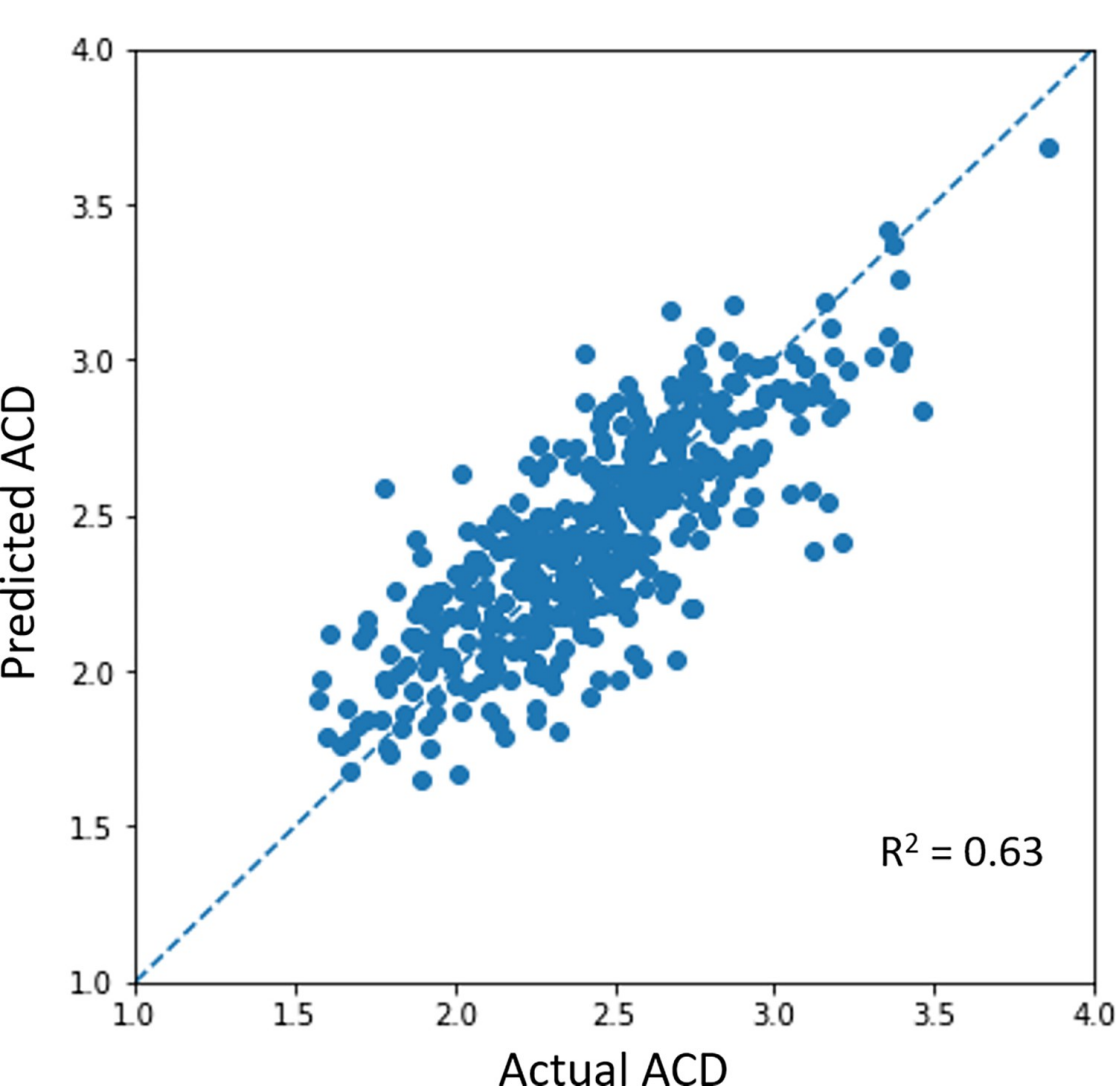
Correlation between actual anterior chamber depth (ACD) measurements and deep learning predicted ACD values in algorithm validation.

**Fig 1** shows the correlation between actual and DL-predicted ACD values in algorithm validation. Our DL algorithm predicted a mean ACD of 2.43 (0.39) mm with a MAE of 0.18 (0.14) mm and R^2^ of 0.63. The MAE of predicted ACD was 0.18 (0.14) mm in eyes with open angles and 0.19 (0.14) mm in eyes with angle closure. When stratified by ACD measurement, we achieved a MAE of 0.20 (0.15) mm for eyes with ACD <2mm (n = 59 eyes); MAE 0.18 (0·13) mm for ACD between 2 to <2.5mm (n = 205 eyes); MAE 0.15 (0.13) mm for ACD ≥2.5 to ≤3mm (n = 166 eyes); MAE 0.31 (0·18) mm for ACD >3mm (n = 32 eyes).

**Fig 2:**
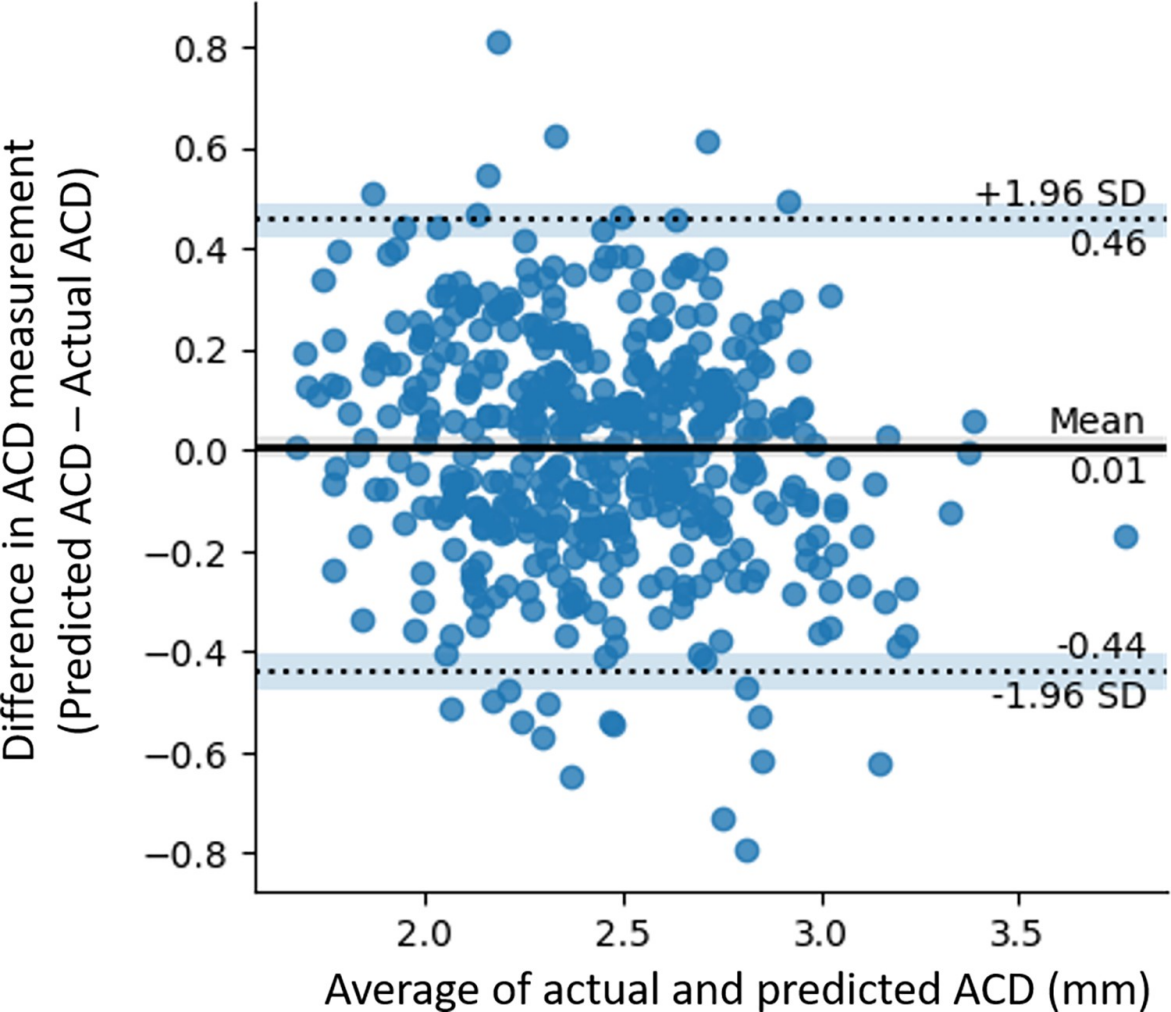
Bland-Altman plot illustrating the agreement between actual and predicted anterior chamber depth (ACD) measurements in algorithm validation.

The Bland-Altman plot showed a mean difference of 0.01mm (Limits-of-agreement [LOA] -0.44, 0.46) between predicted and actual ACD measurements in algorithm validation (**Fig 2**). Although agreement between predicted and actual ACD measurements was good (ICC 0.81, 95% CI 0.77, 0.84), there were 23 observations that were outside the 95% LOA, representing 5% of observations in the test dataset (n = 462 eyes). Systematic bias in DL prediction was insignificant (t-statistics 0.608; P = 0.544) but a weak negative proportional bias was observed (r = -0.17; P <0.01).

**Fig 3:**
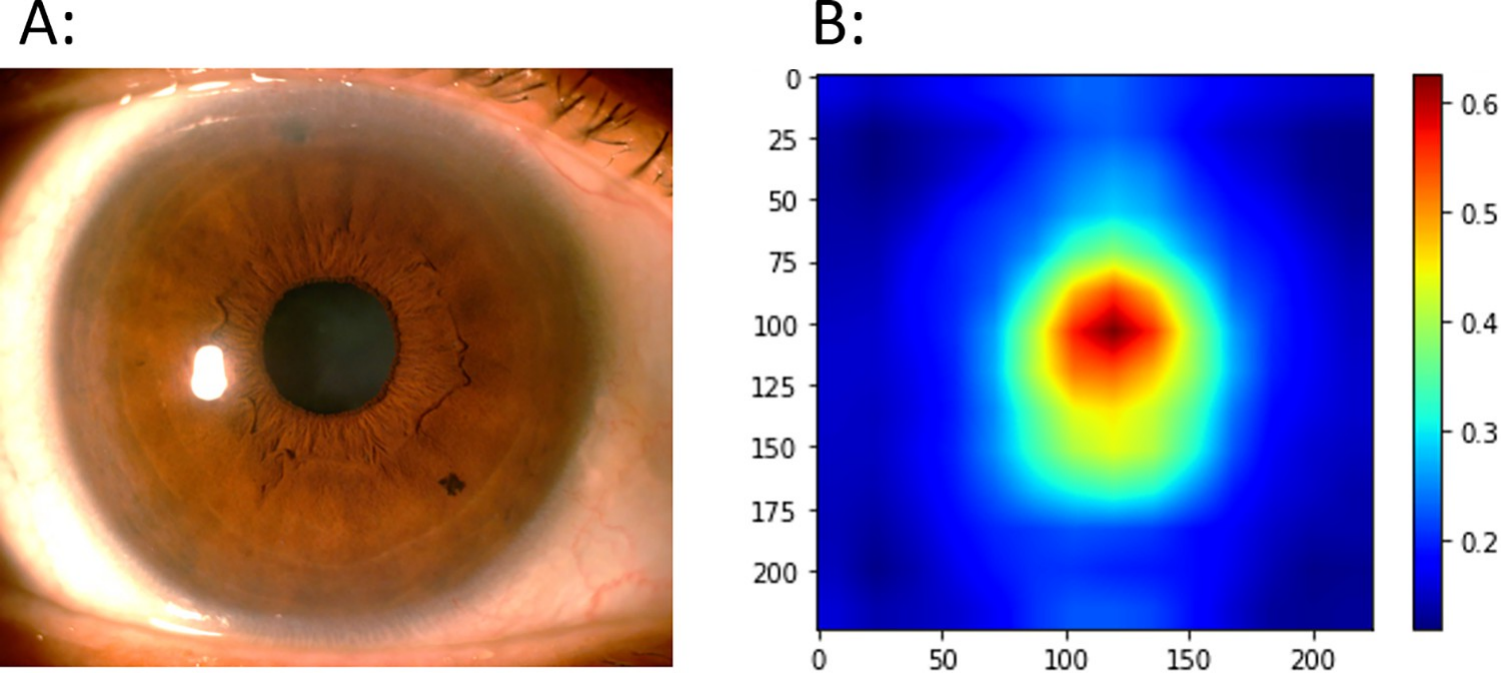
Saliency map illustrating the structural features used by the deep learning algorithm to predict anterior chamber depth in algorithm validation.

In general, our algorithm relied on the pupil and its margins to make its ACD prediction (**Fig 3**). This was similarly observed in eyes with different angle status (**S4 Fig**), different actual ACD measurements (**S5 Fig)**, and amongst the outlier images in our Bland-Altman plot (**S6 Fig**). However, a wider spread, especially in the central red zone, was observed in eyes with ACD >3mm (**D, S5 Fig**) and in the outlier images (**S6 Fig**).

The testing dataset comprised of 257 participants (257 eyes) from SCES and 163 participants (163 eyes) from SINDI (**S2 Table**). Overall, these380 participants (380 eyes) had a mean ACD of 2.58 (0.36) mm, and 28.7% had angle closure (**Table 1**). Our algorithm predicted ACD with a MAE of 0.23 (0.18) mm and R^2^ of 0.37 (**S7 Fig**). The Bland Altman plot showed a mean difference of 0.09mm (LOA -0.47, 0.65) between predicted and actual ACD values (**S8 Fig**) with moderate agreement (ICC 0.57, 95% CI 0.47, 0.66). Likewise, systematic bias (t-statistics 0.597, P = 0.001) and proportional bias was observed (r = -0.39; P <0.01).

## Discussion

In this study, we developed a proof-of-concept DL algorithm that was able to estimate ACD between 0.18 to 0.23mm of actual measurements on average, from anterior segment photographs taken with diffuse illumination. This algorithm mainly utilized the pupil and its margin to make its prediction, which is consistent with ACD measurements derived from ocular biometers. Our study shows that it may be possible for DL to predict depth from 2-dimensional anterior segment photographs, which can be easily acquired without much technical expertise.

In ophthalmology, DL is often applied to posterior segment photographs or OCT scans to detect ocular diseases. [43,44] In contrast, fewer studies have utilized anterior segment photographs to detect anterior eye diseases, including prediction of ACD. [45] Chen et al mounted a smartphone onto a portable slit-lamp device, and utilized a cross-sectional slit-beam to capture anterior segment photographs. [46] Through machine learning, predicted ACD were mostly within 0.20mm of actual measurements, [46] which was similar to our results. Qian et al further developed a DL algorithm to predict shallow ACD (defined as <2.4mm) from Pentacam images and reported an AUC of 0.86 and balanced accuracy of 0.80. [47] Interestingly, Foo et al utilized cycle GAN to predict shallow ACD (defined as ≤2.8mm) from fundus photographs and achieved an AUC of 0.90. [48] Nevertheless, the differences in shallow ACD definition may preclude the generalizability of these algorithms, especially as there is a lack of population-based normative values to guide the categorization of ACD. [28]

Anterior chamber depth may be assessed clinically via slit-lamp bio-microscopy to gain a qualitative (i.e., deep or shallow) or semi-quantitative (e.g., <25%, etc.) appraisal of the risk for angle closure development. However, ACD is a dynamic measurement that changes gradually with age and lens status. [28,49] Thus, the lack of quantitative ACD measurement in settings where ocular biometer or AS-OCT are not readily available may preclude a more nuanced appreciation of its changes over time. For example, ACD has been shown to be inversely correlated with age and increased lens vault. [28,50] This may result in a more “crowded” anterior chamber where structures, such as the iris and lens, are at increased risk of apposition. Importantly, quantitative ACD measurements may further provide investigators with the flexibility of adopting a more granular cut-off for defining “shallow anterior chamber” in the local population. Although the Smith’s technique, first described in 1979, may be used to measure ACD quantitatively over the slit-lamp, [51] the time and expertise required along with the need for manual calculation via a correction factor, may limit its use in community screenings.

Nonetheless, angle closure is a clinically heterogenous disease that requires more than a single anterior chamber parameter for optimal detection. [3] This heterogeneity may explain why there is no single parameter, including ACD, that is optimal to be used in-silos for screening angle closure (**S3 Table**). However, studies showed that a combination of six AS-OCT measurements could identify 95% of angle closure cases with a specificity of 80%, [52] and explain over 80% of variation in quantitative angle width. [53] These quantitative measurements may also predict eyes at higher risk of angle closure development. [54] Thus, future studies should investigate the ability of DL to predict other quantitative measurements that are relevant in the detection and monitoring of angle closure disease from anterior segment photographs, and our proof-of-concept algorithm may serve as a foundation for transfer-learning. In addition, future studies are needed to define the cut-off values that may be used for referral based on these quantitative measurements.

The performance of DL algorithms is influenced by the number of labelled data used in its development. [55] This may be more so when the outcome-of-interest is a continuous variable as opposed to binary or ordinal classification where the number of labels is much fewer. [56,57] In our study, 78.8% of ACD measurements used in training our algorithm were between 2 to 3 mm. Correspondingly, our algorithm was more accurate in predicting ACD values between 2 to 3mm, which highlights the importance of having more data for training, especially those of ACD <2 and >3mm. Although, our algorithm managed to predict ACD within 0.18 to 0.23mm of actual measurements on average by using a relatively small training dataset of only 1,849 images, additional training data are needed to improve the accuracy of prediction given the poorer performances observed in algorithm testing.

Our proof-of-concept study is notable for several results. The use of a quantitative parameter provides us with a more objective reference standard for developing our algorithm. and mitigates against human errors such as image misclassification. [34] Next, saliency maps show that our algorithm mimic an ocular biometer in making its prediction, rather than due to unexplainable or systematic errors. Also, all anterior segment photographs were taken according to the same protocol in both included studies, which reduces variation in data capture. Furthermore, we utilized a diffuse illumination in capturing anterior segment photographs, which is quick to set-up, easier to use, and can be readily replicated.

However, our study is not without its limitations. The performance and generalizability of our algorithm may be improved with larger and more diverse data (e.g., different devices) respectively. Although diffuse illumination was used, our slit beam was tilted at approximately 45 degrees to capture anterior segment images. This causes the light bulb of the slit beam to be reflected at either side of the pupil margin (**S1 Fig**), which may inadvertently create an “illusion of depth”. Thus, future studies may evaluate the use of a straight beam (e.g., through a mobile device) to determine the effect of having the slit beam tilted at an angle. Next, the lack of a fixed fixation target for slit-lamp photography meant that getting good centration was a challenge. Although we excluded images with visibly poor centration, some degree of decentration was inevitable and may influence the accuracy of our algorithm. Also, a weak negative proportional bias was observed where predicted ACD was larger than actual measurements in shallower ACD. This may potentially under-estimate the risk of angle closure.

In conclusion, we developed a proof-of-concept DL algorithm that could predict ACD values from anterior segment photographs. This algorithm mimics an ocular biometer in making its prediction and may serve as a foundation for future work in this area. In addition, it may provide resource scarce settings with a novel tool for monitoring angle closure disease, upon further validation.

## Supporting information

S1 Fig Examples of anterior segment photographs included in this study. Acronym: SiMES, Singapore Malay Eye Study; ISF, Iris Surface Features study; SCES, Singapore Chinese Eye Study.
(TIF)

S2 Fig Schematic diagram of neural network architecture. (TIF)

S3 Fig Distribution of anterior chamber depth in the training dataset. (TIF)

S4 Fig Saliency map illustrating the structural features used by the deep learning algorithm in algorithm validation to predict anterior chamber depth in eyes with different angle status. Footnote: Saliency maps presented above were generated by averaging all saliency maps of eyes with open angles (A; n = 327) and angle closure (B; n = 135).
(TIF)

S5 Fig Saliency maps illustrating the structural features used by the deep learning algorithm in algorithm validation to predict anterior chamber depth in eyes with different categories of actual anterior chamber depth (ACD) measurements. Footnote: Saliency maps presented above were generated by averaging all saliency maps of eyes with actual anterior chamber depth less than <2mm (A; n = 59), 2 to <2.5mm (B; n = 205), ≥2.5 to ≤3mm (C; n = 166); >3mm (D; n = 32).
(TIF)

S6 Fig Saliency maps of predicted ACD values that were outside of the limit-of-agreement in Bland-Altman plot in algorithm validation. Footnote: Saliency maps presented above were generated by averaging all saliency maps of 23 observations that were outside the Limits-of-Agreement in Bland-Altman plot (test dataset). There were 4 observations with actual ACD measurements <2mm, 13 with actual ACD measurements between 2 to 3mm, and 6 with actual ACD measurements >3mm.
(TIF)

S7 Fig Correlation between actual anterior chamber depth (ACD) measurements and predicted ACD values in algorithm testing. (TIF)

S8 Fig Bland-Altman plot illustrating the agreement between actual and predicted anterior chamber depth (ACD) measurements in algorithm testing. (TIF)

S1 Table Current literature on the performance of anterior chamber depth (ACD) in discriminating eyes with angle closure from open angles. * ACD measurement includes central corneal thickness. † Narrow angle diagnosed in eyes with 1) 1 ‘closed’ quadrant (i.e., trabecular meshwork not observed even with indentation gonioscopy) and ≥1 ‘narrow’ quadrants (i.e., trabecular meshwork only observed with indentation gonioscopy) or 2) ≥2 ‘narrow’ quadrants. ‡ Angle closure diagnosed in eyes where the posterior trabecular meshwork (PTM) was not observed in ≥3 quadrants with gonioscopy. § Angle closure diagnosed in eyes where the posterior trabecular meshwork (PTM) was not observed in ≥2 quadrants with gonioscopy. Acronym: ACD, Anterior Chamber Depth; AUC, Area-under-the-curve; PPV, Positive Predictive Value; NPV, Negative Predictive Value; UBM, Ultrasound biomicroscopy
(DOCX)

S2 Table Demographic and ocular characteristics of participants in SEED and the ISF study. *Angle closure was diagnosed in cases where ≥180° posterior trabecular meshwork was not observed with gonioscopy. Acronym: SEED, Singapore Epidemiology of Eye Diseases study; SiMES, Singapore Malay Eye Study; SCES, Singapore Chinese Eye Study; SINDI, Singapore Indian Eye Study; ISF, Iris Surface Features study
(DOCX)

S3 Table Current literature on the performance of different anterior segment optical coherence tomography (AS-OCT) parameters in discriminating eyes with angle closure from open angles. (DOCX)
