# Correction: From 2 dimensions to 3rd dimension: Quantitative prediction of anterior chamber depth from anterior segment photographs via deep-learning

**DOI:** 10.1371/journal.pdig.0000356

**Published:** 2023-09-07

**Authors:** Zhi Da Soh, Yixing Jiang, Sakthi Selvam S/O Ganesan, Menghan Zhou, Monisha Nongiur, Shivani Majithia, Chung Tham, Tyler Hyungtaek Rim, Chaoxu Qian, Victor Koh, Tin Aung, Tien Yin Wong, Xinxing Xu, Yong Liu, Ching-Yu Cheng

There is an error in Supplementary Table 2 and the main text. The work of this article included 380 eyes from 380 participants in the testing dataset. In error, these numbers were presented as 257 from the Singapore Chinese Eye Study (SCES) and 163 from the Singapore Indian Eye Study (line 280–281). (SINDI). However, the correct number is comprised of 229 eyes from (SCES) and 151 eyes from SINDI.

Please find the corrected table below;

**Supplementary Table 2.** Demographic and ocular characteristics of participants in SEED and the ISF study.

	SiMES	SCES	SINDI	ISF study
Participants (N)	943	229	151	343
Age (years)	64.7 (8.1)	61.19 (7.6)	60.35 (6.5)	66.3 (7.5)
Gender (Male, %)	434 (46.0)	104 (45.41)	85 (56.29)	142 (41.4)
Ethnicity (Chinese, %)	0 (0)	229 (100)	0 (0)	316 (92.1)
Eyes (N)	1738	229	151	575
Angle status (%) • Open • Closed*	1627 (93.7) 109 (6.3)	162 (70.7) 67 (29.3)	109 (72.2) 42 (27.8)	42 (7.3) 533 (92.7)
Anterior chamber depth (mm)	2.56 (0.33)	2.56 (0.37)	2.61 (0.34)	2.08 (0.32)

Data presented are mean (standard deviation) for continuous variables; frequency (percentage) for categorical variables

Footnote: *Angle closure was diagnosed in cases where ≥180^0^ posterior trabecular meshwork was not observed with gonioscopy

Acronym: SEED, Singapore Epidemiology of Eye Diseases study; SiMES, Singapore Malay Eye Study; SCES, Singapore Chinese Eye Study; SINDI, Singapore Indian Eye Study; ISF, Iris Surface Features study
