# Supplementary figures and images for: From 2 dimensions to 3rd dimension: Quantitative prediction of anterior chamber depth from anterior segment photographs via deep-learning
Source: PLOS Digit Health. 2023 Feb 1;2(2):e0000193. doi: 10.1371/journal.pdig.0000193 (PMC9931242; doi:10.1371/journal.pdig.0000193)

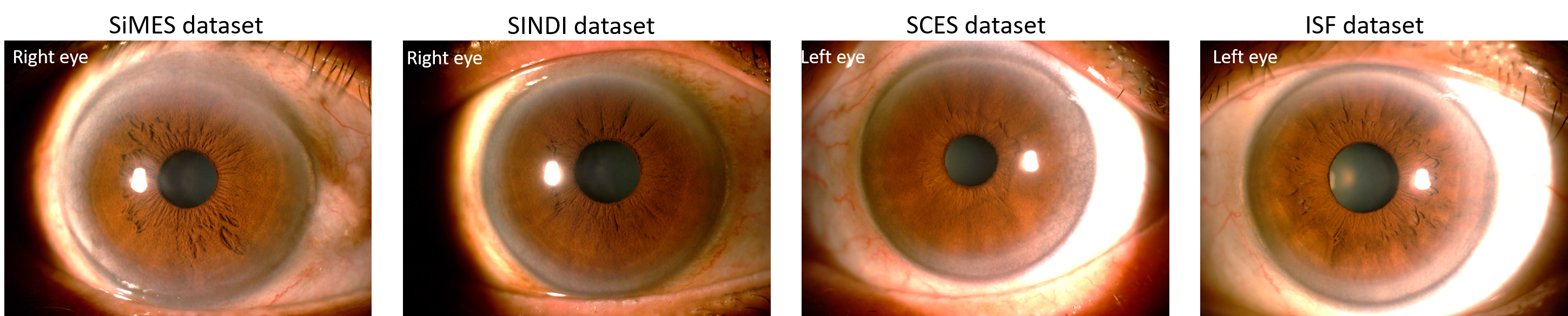

Supplement: S1 Fig — Acronym: SiMES, Singapore Malay Eye Study; ISF, Iris Surface Features study; SCES, Singapore Chinese Eye Study. (TIF) [file pdig.0000193.s001.tif]

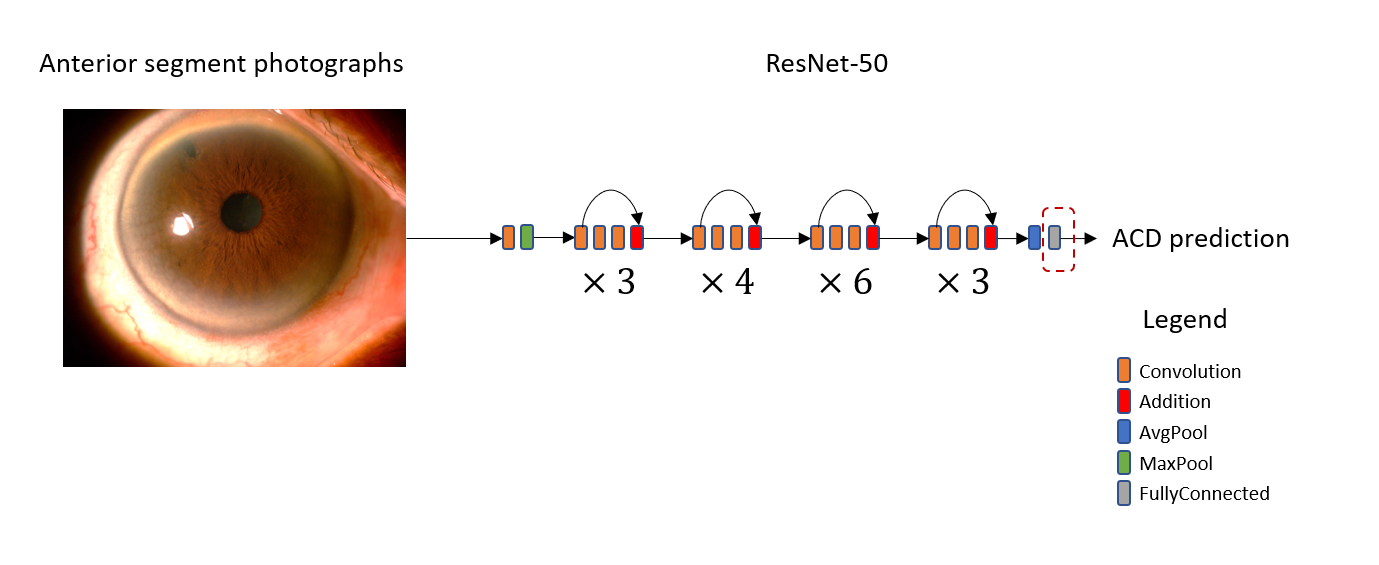

Supplement: S2 Fig — (TIF) [file pdig.0000193.s002.tif]

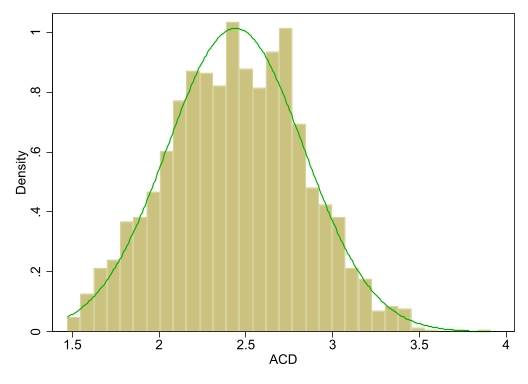

Supplement: S3 Fig — (TIF) [file pdig.0000193.s003.tif]

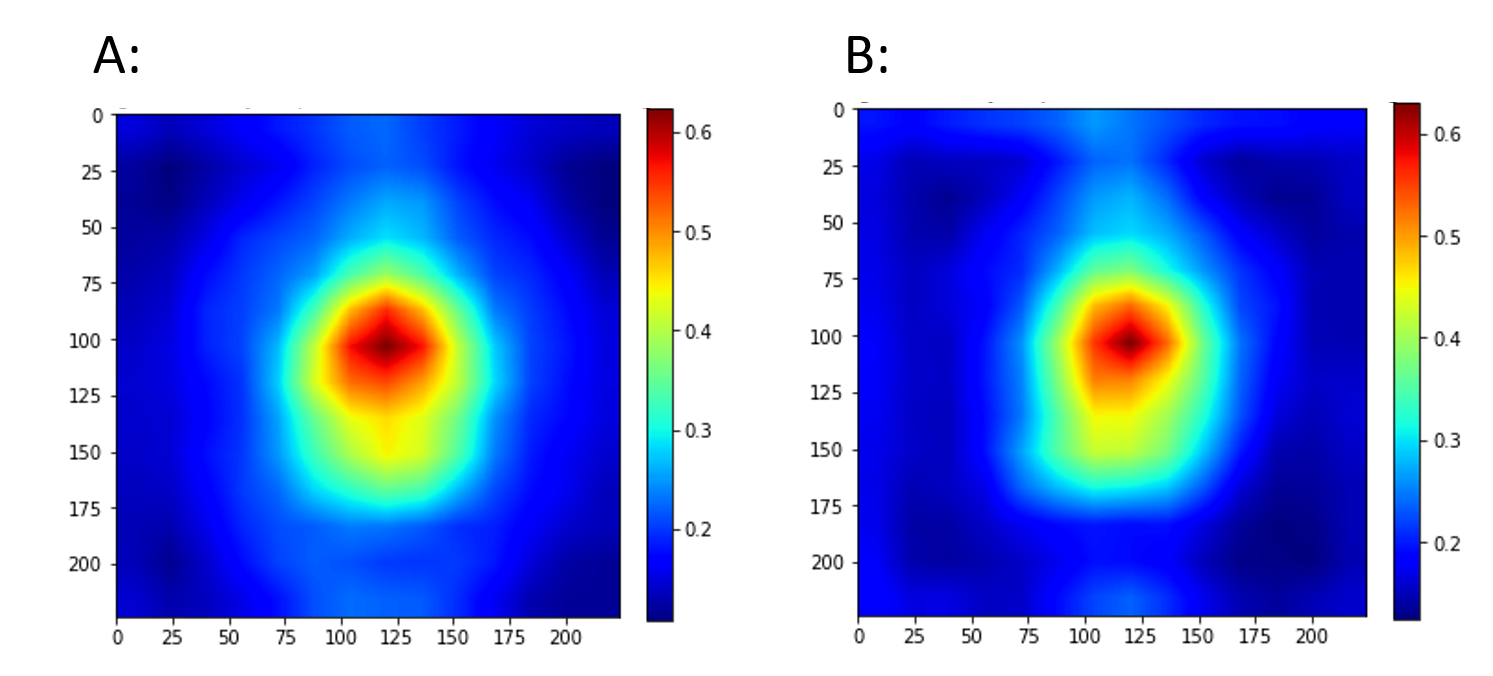

Supplement: S4 Fig — Footnote: Saliency maps presented above were generated by averaging all saliency maps of eyes with open angles (A; n = 327) and angle closure (B; n = 135). (TIF) [file pdig.0000193.s004.tif]

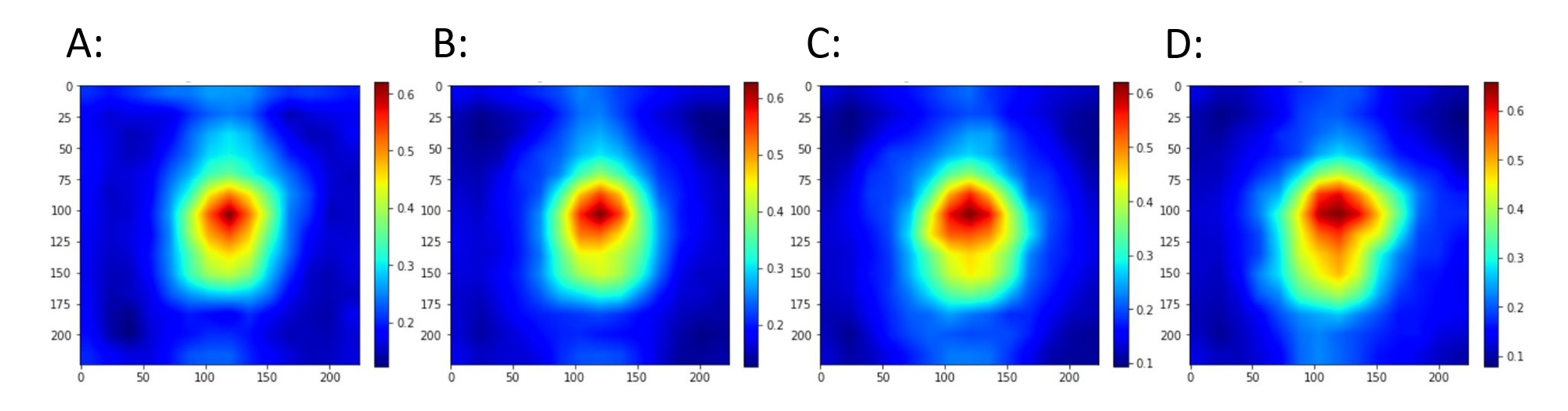

Supplement: S5 Fig — Footnote: Saliency maps presented above were generated by averaging all saliency maps of eyes with actual anterior chamber depth less than <2mm (A; n = 59), 2 to <2.5mm (B; n = 205), ≥2.5 to ≤3mm (C; n = 166); >3mm (D; n = 32). (TIF) [file pdig.0000193.s005.tif]

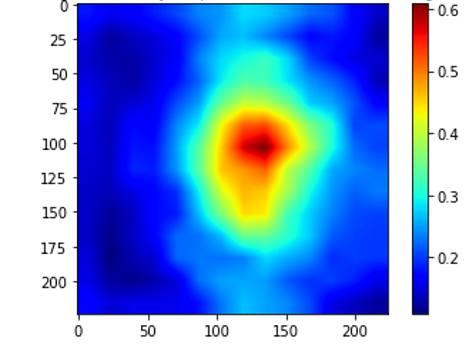

Supplement: S6 Fig — Footnote: Saliency maps presented above were generated by averaging all saliency maps of 23 observations that were outside the Limits-of-Agreement in Bland-Altman plot (test dataset). There were 4 observations with actual ACD measurements <2mm, 13 with actual ACD measurements between 2 to 3mm, and 6 with actual ACD measurements >3mm. (TIF) [file pdig.0000193.s006.tif]

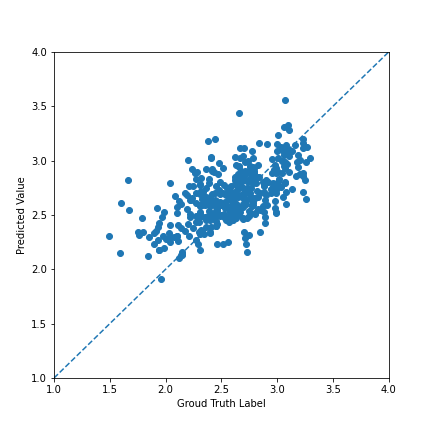

Supplement: S7 Fig — (TIF) [file pdig.0000193.s007.tif]

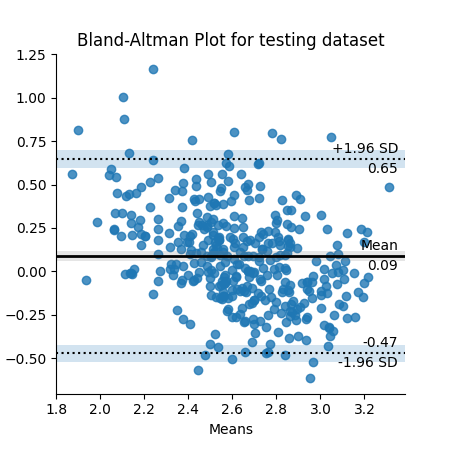

Supplement: S8 Fig — (TIF) [file pdig.0000193.s008.tif]
