# Supplementary material for: From 2 dimensions to 3rd dimension: Quantitative prediction of anterior chamber depth from anterior segment photographs via deep-learning
Source: PLOS Digit Health. 2023 Feb 1;2(2):e0000193. doi: 10.1371/journal.pdig.0000193 (PMC9931242; doi:10.1371/journal.pdig.0000193)
Supplement: S1 Table — * ACD measurement includes central corneal thickness. † Narrow angle diagnosed in eyes with 1) 1 ‘closed’ quadrant (i.e., trabecular meshwork not observed even with indentation gonioscopy) and ≥1 ‘narrow’ quadrants (i.e., trabecular meshwork only observed with indentation gonioscopy) or 2) ≥2 ‘narrow’ quadrants. ‡ Angle closure diagnosed in eyes where the posterior trabecular meshwork (PTM) was not observed in ≥3 quadrants with gonioscopy. § Angle closure diagnosed in eyes where the posterior trabecular meshwork (PTM) was not observed in ≥2 quadrants with gonioscopy. Acronym: ACD, Anterior Chamber Depth; AUC, Area-under-the-curve; PPV, Positive Predictive Value; NPV, Negative Predictive Value; UBM, Ultrasound biomicroscopy (DOCX) [file pdig.0000193.s009.docx]

**Supplementary Table 1. Current literature on the performance of anterior chamber depth in discriminating eyes with angle closure from open angles**

| **Author, year** | **ACD threshold** | **Equipment** | **N (person)** | **AUC** | **Sensitivity** | **Specificity** | **PPV** | **NPV** |
| --- | --- | --- | --- | --- | --- | --- | --- | --- |
| Congdon, 1996 | 2.70mm^†^ | UBM | 562 | - | 76.9 | 87.0 | - | - |
| Devereux, 2000 | <2.22mm^‡^ | UBM | 937 | 0.90 | 83.0 | 81.0 | 24.2 | 98.5 |
| Nolan, 2003 | <2.60mm*^‡^ | UBM | 1090 | 0.81 | 80.2 | 67.2 | 22.5 | 96.6 |
| Nongpiur, 2010 | ≤2.90mm^§^ | IOL Master | 1465 | 0.82 | 80.3 | 84.4 | 54.0 | 95.0 |
| Wu, 2011 | 2.95mm^§^ | IOL Master | 1922 | 0.83 | 76.4 | 88.9 | 42.7 | 97.6 |

* ACD measurement includes central corneal thickness

† Narrow angle diagnosed in eyes with 1) 1 ‘closed’ quadrant (i.e., trabecular meshwork not observed even with indentation gonioscopy) and ≥1 ‘narrow’ quadrants (i.e., trabecular meshwork only observed with indentation gonioscopy) or 2) ≥2 ‘narrow’ quadrants

‡ Angle closure diagnosed in eyes where the posterior trabecular meshwork (PTM) was not observed in ≥3 quadrants with gonioscopy

§ Angle closure diagnosed in eyes where the posterior trabecular meshwork (PTM) was not observed in ≥2 quadrants with gonioscopy

**Acronym**: ACD, Anterior Chamber Depth; AUC, Area-under-the-curve; PPV, Positive Predictive Value; NPV, Negative Predictive Value; UBM, Ultrasound biomicroscopy
