# Supplementary material for: From 2 dimensions to 3rd dimension: Quantitative prediction of anterior chamber depth from anterior segment photographs via deep-learning
Source: PLOS Digit Health. 2023 Feb 1;2(2):e0000193. doi: 10.1371/journal.pdig.0000193 (PMC9931242; doi:10.1371/journal.pdig.0000193)
Supplement: S2 Table — *Angle closure was diagnosed in cases where ≥180° posterior trabecular meshwork was not observed with gonioscopy. Acronym: SEED, Singapore Epidemiology of Eye Diseases study; SiMES, Singapore Malay Eye Study; SCES, Singapore Chinese Eye Study; SINDI, Singapore Indian Eye Study; ISF, Iris Surface Features study (DOCX) [file pdig.0000193.s010.docx]

**Supplementary Table 2. Demographic and ocular characteristics of participants in SEED and the ISF study**

|  | **SiMES** | **SCES** | **SINDI** | **ISF study** |
| --- | --- | --- | --- | --- |
| Participants (N) | 943 | 257 | 163 | 343 |
| Age (years) | 64.7 (8.1) | 61.5 (7.7) | 60.2 (6.6) | 66.3 (7.5) |
| Gender (Male, %) | 434 (46.0) | 123 (47.9) | 91 (55.8) | 142 (41.4) |
| Ethnicity (Chinese, %) | 0 (0) | 257 (100) | 0 (0) | 316 (92.1) |
|  |  |  |  |  |
| Eyes (N) | 1738 | 257 | 163 | 575 |
| Angle status (%)   - Open - Closed* | 1627 (93.7)  109 (6.3) | 185 (72.0)  72 (28.0) | 116 (71.2)  47 (28.8) | 42 (7.3)  533 (92.7) |
| Anterior chamber depth (mm) | 2.56 (0.33) | 2.61 (0.39) | 2.64 (0.36) | 2.08 (0.32) |

Data presented are mean (standard deviation) for continuous variables; frequency (percentage) for categorical variables

Footnote: *Angle closure was diagnosed in cases where ≥180^0^ posterior trabecular meshwork was not observed with gonioscopy

Acronym: SEED, Singapore Epidemiology of Eye Diseases study; SiMES, Singapore Malay Eye Study; SCES, Singapore Chinese Eye Study; SINDI, Singapore Indian Eye Study; ISF, Iris Surface Features study
