# Supplementary material for: From 2 dimensions to 3rd dimension: Quantitative prediction of anterior chamber depth from anterior segment photographs via deep-learning
Source: PLOS Digit Health. 2023 Feb 1;2(2):e0000193. doi: 10.1371/journal.pdig.0000193 (PMC9931242; doi:10.1371/journal.pdig.0000193)
Supplement: S3 Table — (DOCX) [file pdig.0000193.s011.docx]

**Supplementary Table 3. Current literature on the performance of different anterior segment optical coherence tomography (AS-OCT) parameters in discriminating eyes with angle closure from open angles**

| **Author, year** | **Variable, cut-off** | **AUC** | **Specificity** | **Sensitivity** | **PPV** | **NPV** |
| --- | --- | --- | --- | --- | --- | --- |
| Narayanaswamy, 2016 | AOD500, ≤0.177 | 0.81 | 76.1 | 85.1 | 28.3 | 97.8 |
|  | AOD500, ≤0.191 | 0.82 | 74.6 | 88.9 | 27.9 | 98.3 |
|  | AOD750, ≤0.225 | 0.83 | 84 | 82.5 | 36.4 | 97.7 |
|  | AOD750, ≤0.258 | 0.84 | 77.4 | 90.2 | 30.6 | 98.6 |
|  | TISA500, ≤0.076 | 0.74 | 75.2 | 73.3 | 24.6 | 96.1 |
|  | TISA500, ≤0.103 | 0.74 | 59.1 | 88.2 | 19.3 | 97.7 |
|  | TISA750, ≤0.134 | 0.79 | 77.5 | 80.3 | 28.4 | 97.2 |
|  | TISA750, ≤0.151 | 0.80 | 76.7 | 83.5 | 28.4 | 97.6 |
| Nongpiur, 2010 | ACW, ≤11.7 | 0.63 | 60.5 | 64.4 | 30.9 | 86.2 |
| Wu, 2011 | ACA, 17.9 | 0.88 | 90.2 | 89.9 | 55.1 | 97.7 |
| Wu, 2011 | ACV, 116 | 0.88 | 85.1 | 90.2 | 54.4 | 97.8 |
| Tan, 2012 | LV, ≥0.576 | 0.82 | 77.5 | 85.7 | 51 | 95.2 |

Footnote: Angle closure was defined as posterior trabecular meshwork of ≥180^0^ not observed on gonioscopy

Acronym: AUC, Area Under the Curve; PPV, Positive Predictive Value; NPV, Negative Predictive Value; AOD, Angle Opening Distance; TISA, Trabecular Iris Space Area; ACD, Anterior Chamber Depth; ACW, Anterior Chamber Width; ACA, Anterior Chamber Area; ACV, Anterior Chamber Volume; LV, Lens Vault.
